# CLINICAL AND EPIDEMIOLOGICAL CHARACTERISTICS AND SURVIVAL OUTCOMES OF CHILDREN WITH NEUROBLASTOMA: 21 YEARS OF EXPERIENCE AT THE *INSTITUTO DE ONCOLOGIA PEDIÁTRICA*, IN SÃO PAULO, BRAZIL

**DOI:** 10.1590/1984-0462/;2018;36;3;00007

**Published:** 2018

**Authors:** Januária Nunes Lucena, Maria Teresa Seixas Alves, Simone Campos Vieira Abib, Gabriel Oliveira de Souza, Regina Pukenis de Castro Neves, Eliana Maria Monteiro Caran

**Affiliations:** a Universidade Federal de São Paulo, São Paulo, SP, Brasil.

**Keywords:** Neuroblastoma, Child, Epidemiology, Neuroblastoma, Criança, Epidemiologia

## Abstract

**Objective:**

To describe the clinical and epidemiological characteristics and survival outcomes of children with neuroblastoma (NB) treated at a pediatric oncology center from 1991 to 2012.

**Methods:**

A retrospective study with clinical and epidemiological data from 258 patients with neuroblastoma treated at a pediatric oncology center from 1991 to 2012, using medical records.

**Results:**

The average age of the children at diagnosis was 40.5±46.4 months with a median age of 28.9 months (interquartile range 42.2). The male:female ratio was 1.3:1, and 1% of the patients were asymptomatic. The most frequent manifestations were: fever (25%), abdominal pain (22%), abdominal mass (19%), and bone pain (19%). The mean time from symptom onset to diagnosis was 3.0±4.8 months. The most common location of the tumor was the abdomen (63%). Metastases occurred in the bone marrow (37%) and in the bone (33%). Overall survival (OS) and event-free survival (EFS) in five years were 62 and 52%, respectively. The main cause of death was the progression of the disease (72%).

**Conclusions:**

The clinical features of children with neuroblastoma are variable and mostly nonspecific, which makes clinical recognition difficult and, in general, too late. In children less than 5 years old, with an abdominal mass and/or bone pain, irritability, and a fever from an unknown cause, neuroblastoma should be considered as a possible diagnosis.

## RESUMO

**Objetivo:**

Descrever as características clínicas e epidemiológicas e a sobrevida de crianças com neuroblastoma (NB) atendidas em centro de oncologia pediátrica, no período de 1991 a 2012.

**Métodos:**

Estudo retrospectivo, com análise de dados clínicos e epidemiológicos de 258 pacientes com NB atendidos em centro de oncologia pediátrica, no período de 1991 a 2012, por meio de consulta a prontuários médicos.

**Resultados:**

A idade média das crianças foi de 40,5±46,4 meses, e a mediana, de 28,9 meses (intervalo interquartil 42,2); relação masculino:feminino 1,3:1, sendo 1% dos pacientes assintomáticos. As manifestações mais frequentes foram: febre (25%), dor abdominal (22%), massa abdominal (19%) e dor óssea (19%). O tempo médio do início dos sintomas até a realização do diagnóstico foi de 3,0±4,8 meses. A localização do tumor mais frequente foi o abdome (63%). As metástases ocorreram na medula óssea (37%) e nos ossos (33%). A sobrevida global (SG) e a sobrevida livre de eventos (SLE) em cinco anos foram de 62 e 52%, respectivamente. A principal causa de óbito foi a progressão da doença (72%).

**Conclusões:**

As características clínicas das crianças com NB são variáveis e, em sua maioria, inespecíficas, o que torna o reconhecimento clínico difícil e, em geral, tardio. Em crianças com idade inferior a 5 anos, massa abdominal e/ou dor óssea, irritabilidade e febre de origem indeterminada, o diagnóstico de NB deve ser considerado.

## INTRODUCTION

Neuroblastoma (NB) is the most common malignant extracranial solid tumor found in children, and it is the most common cancer to be diagnosed in the first year of a child’s life.^1^ ^,^ ^2^ ^,^ ^3^ ^,^ ^4^ ^,^ ^5^ ^,^ ^6^ The annual incidence of the disease is 10.5 per one million children under the age of 15, and there around 700 new cases per year in the United States.^1^ ^,^ ^2^ ^,^ ^4^ ^,^ ^5^ ^,^ ^7^ In Brazil, from a study based on an analysis of the population records for cancer, which included 12 cities and the Federal District, the incidence of NB was 5.9 per one million inhabitants under the age of 15. Nevertheless, further studies that include the entire country are required in order to make definitive conclusions.^8^

NB occurs most commonly in children that are younger than 5 years of age, and there is a small predominance of it in males. ^1^ ^,^ ^6^ ^,^ ^9^ The cancer can be located anywhere along the sympathetic chain ganglia, including the paravertebral and posterior mediastinal regions, but it is mainly found in the medullary region of the adrenal gland.^1^ ^,^ ^10^ ^12^ This embryonic tumor, which is derived from precursor cells of the sympathetic nervous system, is an important challenge for health professionals, since it is associated with 15% of pediatric oncology-related mortality.^4^ ^,^ ^13^

One of the main features of NB is the various ways it presents itself, which depends on numerous factors, such as patient’s age, the location of the tumor, the stage, the presence of metastases, and paraneoplastic syndromes.^1^ ^,^ ^10^ The main signs and symptoms, which are usually delayed in appearing, are often non-specific and similar to other childhood diseases, making early diagnosis difficult for pediatricians. Signs of the disease vary from a painless mass that is detected accidentally, to a rapidly growing progressive tumor.^10^ Patients with a disease that is localized may be asymptomatic, and therefore, may be diagnosed for other conditions that are not related to the tumor during a medical examination.^1^ Classical symptoms such as fever, pain, weight loss and irritability are associated with metastatic NB.^1^ ^,^ ^10^ ^,^ ^11^ Proptosis and periorbital bruising are frequent and result from the way the tumor infiltrates the periorbital bones.^11^

Currently, the choice of therapy depends on where the patient is stratified in the risk groups.^12^ ^,^ ^14^ ^,^ ^15^ Patients at low or intermediate risk, and with favorable biological tumor characteristics have high survival rates. However, despite advancements in molecular biology and treatment strategies, high-risk patients still have a very poor prognosis.^3^ ^,^ ^15^ ^18^

Considering the scarcity of publications with significant cases in Brazil, and the difficulty of clinical diagnosis of NB, we conducted this study with the objective of evaluating the clinical and epidemiological characteristics and the survival outcomes of children with NB in a pediatric oncology center.

## METHOD

This was a retrospective study with an analysis of the clinical and epidemiological data of 263 patients with NB admitted to the center from 1991 to 2012. Information was obtained through the analysis of medical records. Three patients with incomplete information were excluded, in addition to two that were admitted for palliative care only. As such, 258 cases were included in the study.

NB was diagnosed either by means of a biopsy and an anatomopathological examination, or through the study of bone marrow that had been infiltrated by the tumor, which is associated with the presence of catecholamine metabolites in the urine, according to international criteria.^1^ ^,^ ^11^

**Chart 1:**
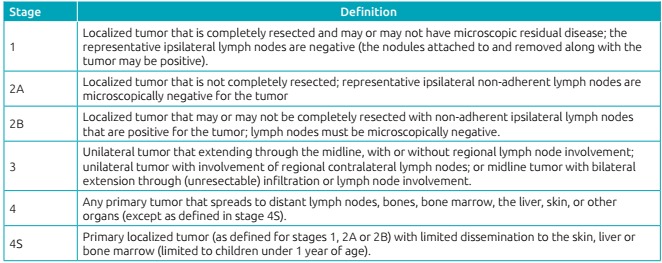
International Neuroblastoma Staging System^1^.

Patients were classified according to the criteria from International Neuroblastoma Staging System (INSS) into stages 1, 2, 3, 4 and 4S (Chart 1).^1^

The World Health Organization’s (WHO) histological classification was used.^1^ In the cytogenetic evaluation of the neoplastic cell, the MYCN oncogene was investigated using the florescent in situ hybridization method (FISH), and was considered to be amplified when it was above ten copies.^19^

Overall survival (OS) was considered to be the time that elapsed from the diagnosis to death or to the final evaluation. As such, children with or without an active disease were included in this group. Event-free survival (EFS) was defined as the time that elapsed from diagnosis to relapse, death, or the final evaluation, and thus part of this group included children with no active disease.

A literature review was conducted by searching the PubMed, the Medical Literature Analysis and Retrieval System Online (MEDLINE) and the Latin American and Caribbean Literature in Health Sciences (LILACS) databases, using the terms “neuroblastoma”, “neuroblastoma Brazil” and “embryonal tumors”, from 2000 to 2017.

The categorical variables were expressed in frequency and percentage, and the numerical variables were expressed using averages, standard deviations, minimums, medians, maximums and valid observation totals. To compare gender versus stage, and age versus stage, the likelihood ratio test was used. To compare stage versus relapse, Pearson’s chi test was applied. The comparison of medical history time versus stage time was done using the analysis of variance (ANOVA) model. Multiple comparisons were performed using the Bonferroni test. To perform the survival analyzes, the Kaplan Meier test was used. In all of the analyses, a p-value = 0.05 was considered significant.

This study was approved by the Research Ethics Committee of the Universidade Federal de São Paulo (UNIFESP), under registration number 1,668/11.

## RESULTS

The age at the time of diagnosis of the 258 patients with NB ranged from 4 days old to 30 years old, with a mean of 40.5 ± 46.4 months and a median of 28.9 months (and an interquartile range of 42.2 months). Children aged 1 to 4 years old made up the largest group (49%), followed by children under 1 year of age (29%), children aged 5 to 9 years old (17%) and, in the lowest percentage, those 10 years of age or older (5%). Of the patients, 148 (57%) were male, and 110 (43%) were female. There was a predominance of males and a ratio of 1.3:1.1, males to females. With regard to the patient’s age, it is worth mentioning that, since NB is a tumor that predominantly occurs in childhood, 90% of the cases found were in children under 10 years of age. The clinical management of these patients and the development of therapeutic protocols are not part of the daily practice of adult oncologists. As such, even older patients outside the pediatric age group may be referred to pediatric oncologists that have more experience with this pathology.

With regard to where the tumor was located, 164 (63.0%) cases were found in the abdomen, 49.0% in the adrenal region and 15.0% in the retroperitoneal region. The left adrenal (61.0%) was more affected than the right one (39.0%), and the other sites were: the paravertebral region (22.0%), the mediastinum region (12.0%), the cervical region (2.0%), an undetermined region (0.4%) and other sites (0.4%).

The most frequent signs and symptoms were fever (25.0%), abdominal pain (22.0%), abdominal mass (20.0%) and bone pain (20.0%) (Table 1). Of the patients included in the study, 3 (1.0%) had Horner’s syndrome, 5 (2.0%) had Pepper’s syndrome, and 4 (1.0%) had opsomyoclonus / ataxia. The most common sites of metastasis were the bone marrow (37.0%), the bones (33.0%), the lymph nodes (13.0%), the liver (10.0%), the skin (0.4%) and other locations (5.0%). Of the 258 patients, 86 (33.0%) had metastases in more than one site.

**Table 1:** Distribution of the signs and symptoms in 258 patients with neuroblastoma.

Signs or symptoms	Total number of patients
Fever	64 (25%)
Abdominal pain	58 (22%)
Abdominal mass	54 (21%)
Bone pain	50 (19%)
Abdominal distension	46 (18%)
Weight loss	39 (15%)
Neurological change	28 (11%)
Bruising	16 (6%)
Nodule on the skull	10 (4%)
Adenomegaly	7 (3%)
Systemic arterial hypertension	5 (2%)
Others	104 (40%)

Asymptomatic patients accounted for 13% of the cases. Their tumors were detected when an abdominal mass was touched during a routine consultation or after being examined because of other complaints that were unrelated to the tumor. According to the INSS stages, the majority of these patients had a localized disease: 46% were in stage 1; 6% were in stage 2; 27% were in stage 3; 12% were in stage 4; and 9% were in stage 4S. In the asymptomatic patients, the most frequent tumor site was the abdominal region (43%) - 34% in the suprarenal and 9% in the retroperitoneal region - followed by the mediastinum region (36%) and the paravertebral region (21%).

The mean time from the onset of symptoms to the diagnosis was 3.0 ± 4.8 months, with a variation of 0 to 32 months. There was no statistical difference between the patient’s stage and the time from onset of the symptoms. The median time among the onset of symptoms and diagnosis in all of the stages was very close (p = 0.118) (Table 2).

**Table 2:** Comparison between staging and time from onset of signs and symptoms (months) using the non-parametric Kruskal Wallis test.

Time from onset of signs and symptoms (months)	Stage 1	Stage 2	Stage 3	Stage 4	Stage 4S	p-value
Average±standard deviation	3.1 ± 6.0	6.0±9.8	3.2±4.9	2.7±3.8	0.8±1.1	0,118
Median (minimum-maximum)	1.0 (0-27)	1.5 (0-32)	1.0 (0-24)	2.0 (0-24)	1.0 (0-4)	
Total	22	10	61	111	11	

The diagnosis of NB was made by performing a tumor biopsy and an anatomopathological examination of the 202 (78%) patients. Furthermore, the diagnosis was made based on the study of bone marrow infiltrated by the tumor, which is associated with the presence of catecholamine metabolites in urine in 56 (22%) patients. According to the INSS stages, it was observed that: 38 (15%) patients were in stage 1; 12 (5%) were in stage 2; 74 (29%) were in stage 3; 120 (46%) were in stage 4; and 14 (5%) were in stage 4S.

The MYCN amplification study was performed in 44 (17%) patients and was inconclusive for four of them. Ten patients (25%) had MYCN amplification, while 30 had no such alteration. Most patients (90%) with MYCN amplification showed that the disease was advanced at the time of diagnosis (stages 3 and 4). Only one patient with a localized disease had MYCN amplification.

Of the 258 patients analyzed, 171 (66%) entered into remission. Of these, 57 (33%) had tumor recurrence, 17 (30%) had local recurrences, 35 (61%) were further away, and five (9%) had local and distant recurrences. Patients with a metastatic disease at the time of diagnosis had more relapses (69% of the total) (p = 0.002). In stages 1, 2 and 4S, there were two cases of relapse in each group. The two stage 1 patients who relapsed were older than 1 year old at the time of diagnosis, and tumor recurrence occurred at the primary site (abdomen). The recurrence time ranged from 3 to 84 months, with an average of 18.2 months and a median of 11.5 months. The most frequent sites were: bone marrow (47%), bones (45%), lymph nodes (7%), lungs (2%) and the central nervous system (2%).

The OS of the patients studied was 62% in five years, and 53% in ten years. EFS was 52% in 5 years and 47% in 10 years, both of which were calculated using the Kaplan Meier method. The main cause of death was disease progression (72%). Death due to toxicity from chemotherapy occurred in 22 (23%) patients. Death due to surgical complications was observed in 4 (4%) cases. The mean follow-up time was 58 months.

**Graph 1:**
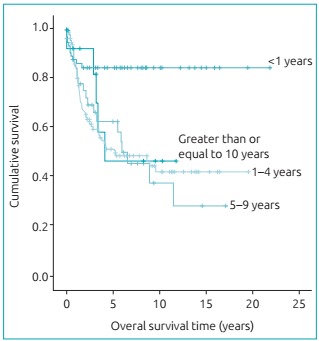
Overall survival curves by age range (Kaplan-Meier; p-value=0.001).

When analyzing the survival curves for the different age groups, it was observed that patients less than 1 year old had a longer duration of OS and EFS than those of other groups, which did not show any difference (p = 0.001) (Graph 1). Patients younger than 1 year old had an advanced disease at the time of diagnosis (38% were stage 3, and 22% were stage 4). The tumor was most frequently located in the adrenal region (40%), followed by the paravertebral (24%), retroperitoneal (18%), mediastinum (15%) and cervical (3%) regions.

**Graph 2:**
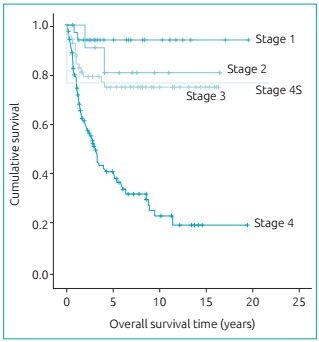
Overall survival curves by stage (Kaplan-Meier; p-value<0.001).

Statistical analysis showed a statistically significant difference (p <0.05) with respect to stage 4, when comparing SF and SLE, and had survival rates that lower than the other stages, which did not differ significantly (Graph 2).

## DISCUSSION

Recognizing NB signs and symptoms is not always easy, because of the low incidence of the disease and because of the fact that other common childhood diseases have a very similar clinical picture.^20^ Knowing NB’s epidemiological characteristics, such as its prominence in childhood, in addition to anamnesis and a physical examination, may cause medical professionals to suspect the presence of the tumor.^1^ ^,^ ^11^ ^,^ ^12^ ^,^ ^13^ ^,^ ^21^ ^,^ ^22^ ^,^ ^23^ ^,^ ^24^ ^,^ ^25^

According to the literature, the average age of children with NB at the time of diagnosis is 23 months old. A review conducted in the United States of 3,666 children with NB, in cooperative groups between 1986 and 2001, showed evidence of a mean age of 19 months old.^11^ In our cases, the average age was 40.5 months old, which could reflect the delay in diagnosis in the majority of the patients studied. Interestingly, 28% of the patients were younger than 1 year old, which confirms the high incidence of the tumor in this age group.

The children with NB had an extremely varied diagnostic clinical picture, which is similar to what has been described in the literature.^26^ In our study, non-specific, systemic signs, such as fever (25%) and weight loss (15%), were frequently reported. These signs and symptoms were also prevalent in the study performed by Collaço et al., who analyzed 50 cases of NB in a reference hospital in Curitiba, Paraná.^26^

In addition to the systemic signs, the patients presented specific symptoms based on the location of the tumor. In 63% of the cases, NB occurred in the abdomen, which frequently ended up compromising the adrenal gland. In those cases, the signs included distension, pain, and a palpable abdominal mass. With regard to the physical examination, the tumor, which is hard, fixed and difficult to demarcate, is situated in the kidneys and grows into the hypochondrium and the flank, and is able to exceed the midline of the abdomen.^11^

It was common to find the tumor in the paravertebral region (22%). In this location, NB tends to extend through the neural foramen, compress the spinal cord, and trigger neurological signs and symptoms: radicular pain, paraplegia, and fecal/urinary incontinence. Spinal cord compression is quite dangerous and needs to be dealt with as quickly as possible, because, if prolonged, it can cause permanent neurological damage. In this study, 37% of the patients with paravertebral tumors had symptoms associated with spinal cord compression.

At the time of diagnosis, 46% of the patients had distant metastases (stage 4), especially in the bone marrow and in the skeletal system. Not infrequently, patients seek out a doctor with complaints due to the metastases: diffuse bone pain, anemia, pancytopenia, irritability, ocular proptosis, periorbitary bruising, nodules in the skull, etc. The bone pain can be intense and prevent the child from moving freely.

The percentage of asymptomatic patients was 13%, and their diagnosis was made when a doctor touched a mass in their abdomen during a routine exam or after analyzing imaging tests that were performed to investigate other non-tumor related complaints. These “accidental” findings are common in tumors located in the mediastinum and in small tumors in the abdomen. The presence of an asymptomatic abdominal mass stresses the need and the importance of performing careful physical examinations in order to detect tumors in their early stages.

In NB, the incidence of characteristic symptoms is low. Only 12 (5%) patients had signs and symptoms of paraneoplastic or clinical syndromes associated with NB. Opsomyoclonus / ataxia, which four (1%) children had in this study, is linked to neuroblastic tumors in 50% of cases.^21^ It is a paraneoplastic syndrome that is characterized by ataxia (lack of coordination with regard to muscle movements), myoclonus (sudden muscle contractions), opsoclonus (involuntary, multidirectional, uncoordinated and hyperkinetic ocular movements) and irritability, with an incidence of 4.0% reported in the literature. Other clinical syndromes, such as intractable diarrhea, which may occur when neoplastic cells produce vasoactive intestinal peptide (VIP), and Horner’s syndrome, which is characterized by unilateral ptosis, miosis and anhidrosis (usually associated with tumors in the upper thoracic or cervical region), also have a low incidence - 3.1 and 2.0%, respectively.^21^ In infants with stage 4S tumors, the liver is commonly involved to a large extent, which can lead to a respiratory failure characterized as Pepper’s syndrome, observed in 29% of the stage 4S patients in this study.^1^ ^,^ ^11^

Although NB is a catecholamine producer in 90% of all cases, tachycardia, sweating and hypertension are rare. It should be emphasized that blood pressure should always be measured at the time of diagnosis and during the follow-up for these patients.

The average time taken to diagnose patients with NB varies in accordance with different pediatric oncology centers. The Royal Hospital for Sick Children in Edinburgh showed an average time of 5.3 weeks. The cooperative group called the Pediatric Oncology Group in the United States showed an average time of 5.4 weeks. And the Hospital do Câncer de São Paulo showed an average time of 18.6 weeks.^20^ In our study, average time from symptom onset to diagnosis was 12 weeks, but there was no difference between symptom time and stage assessment of the patients (p = 0.118), which does not appear to have influenced survival. A study conducted by Parise et al., who, over 11 years, evaluated 125 patients with NB from three pediatric oncology hospitals in the State of Paraná, concluded that diagnosis was delayed and, consequently, there were relatively low survival rates.^13^ These findings may reflect the biological heterogeneity of NB, which may explain the presence of a localized disease in patients with prolonged symptoms and a delayed diagnosis.

When abdominal or pelvic NB is suspected, an ultrasound is usually the first imaging test to be performed. However, for better delimitation of the tumor, other tests, such as a tomography or resonance test, are necessary. A resonance test is critical, especially in paravertebral tumors, in order to evaluate spinal cord compression. Other diagnostic or staging exams include: urinary catecholamines, metaiodobenzylguanidine (MIBG) mapping, a bone marrow biopsy, a myelogram, and a cytogenetic evaluation of the neoplastic cell.^27^

In relation to INSS staging, which is based on the patient’s age, the extent of the disease and tumor resection, the present study showed a predominance (75% of cases) of patients with a disease in its advanced stages (stages 3 and 4). In these stages, there was a greater percentage of patients from 1 to 4 years old (p <0.05). Several epidemiological studies show similar results, with an incidence of patients with an advanced form of the disease (local or disseminated) greater than 60%. ^6^ ^,^ ^13^ ^,^ ^22^ ^25^ These findings reflect the aggressive behavior of NB with regard to early metastases. On the other hand, the stage of the child’s NB has a relevant influence on the prognosis, as shown in Chart 1. The survival of patients in the initial stages (stages 1 and 2) and stage 4S presented a statistically significant difference (p<0.05) in relation to stage 4, which has lower survival rates (SG of 41% and SLE of 26% in 5 years).

Children with NB are stratified as having low, intermediate and high risk NB recurrence.^1^ This classification depends on innumerable clinical prognostic (age, stage, tumor location, serum levels of lactic dehydrogenase and ferritin) and biological (histopathological and cytogenetic classification) factors. Factors associated with a worse prognosis include age greater than 1 year old, a metastatic disease at the time diagnosis and an unfavorable histopathological classification, according to Shimada’s classification.^2^ The patient’s age is an independent prognostic factor. Patients younger than 1 year of age at the time of diagnosis present better odds of survival than older children.^1^ ^,^ ^11^ In our study, these patients presented longer OS and EFS than the others (p = 0.01), a result similar to that in the international literature.

The MYCN oncogene is an important prognostic factor in NB, as it is associated with aggressive behavior and an unfavorable outcome.^2^ ^,^ ^4^ It is amplified in 25 to 35% of the NBs, and in about 30 to 40% of the cases dealing with stage 3 and 4 patients. ^1^ ^,^ ^14^ ^,^ ^28^ In our study, MYCN oncogene amplification was performed in 44 (17%) patients, and 10 (25%) presented MYCN amplification, of which 90% had an advanced disease at the time of diagnosis (stages 3 and 4).

The intensity of the treatment and therapeutic planning (including the need for a bone marrow transplantation) depend on the risk classification. While patients at low and intermediate risk have survival rates above 90%, those at high risk have a poor prognosis.^3^ The OS of the patients studied was 62% in five years, reflecting the high percentage of patients diagnosed with an advanced disease.

Currently, many studies with molecular targeting (anti MYCN, anaplastic lymphoma kinase - ALK, phosphatidylinositol 3 kinase / serine threonine kinase inhibitors - PI3K target of rapamycin - mTOR target protein and aurora kinase) and immunotherapy are currently being developed for the treatment of high-risk NB. They show promising but incipient results.^29^

The clinical and epidemiological characteristics of children with NB who were assisted at our center from the period of 1991 to 2012 were similar to those described in the literature. The clinical diagnosis of NB is difficult, since its manifestations are variable and nonspecific. However, in children under 5 years old, with an abdominal mass and / or bone pain, irritability, fever from an unknown cause, the diagnosis of NB should be considered.
